# Identification of risk factors for postoperative pulmonary complications in general surgery patients in a low-middle income country

**DOI:** 10.1371/journal.pone.0274749

**Published:** 2022-10-11

**Authors:** Katelyn Morris, Kylie Weston, Alyssa Davy, Susan Silva, Victoria Goode, Katherine Pereira, Petra Brysiewicz, John Bruce, Damian Clarke

**Affiliations:** 1 School of Nursing, Duke University, Durham, North Carolina, United States of America; 2 School of Nursing and Public Health, University of KwaZulu-Natal, Durban, South Africa; 3 Pietermaritzburg Metropolitan Trauma Service, Grey’s Hospital, Pietermaritzburg, South Africa; 4 Department of Surgery, University of KwaZulu-Natal, Durban, South Africa; Stanford University School of Medicine, UNITED STATES

## Abstract

**Background:**

Postoperative pulmonary complications (PPCs) are an important cause of perioperative morbidity and mortality. Although risk factors for PPCs have been identified in high-income countries, less is known about PPCs and their risk factors in low- and middle-income countries, such as South Africa. This study examined the incidence of PPCs and their associated risk factors among general surgery patients in a public hospital in the province of KwaZulu-Natal, South Africa to inform future quality improvement initiatives to decrease PPCs in this clinical population.

**Methods:**

A retrospective secondary analysis of adult patients with general surgery admissions from January 1, 2013 to December 31, 2017 was conducted using data from the health system’s Hybrid Electronic Medical Registry. The sample was comprised of 5352 general surgery hospitalizations. PPCs included pneumonia, atelectasis, acute respiratory distress syndrome, pulmonary edema, pulmonary embolism, prolonged ventilation, hemothorax, pneumothorax, and other respiratory morbidity which encompassed empyema, aspiration, pleural effusion, bronchopleural fistula, and lower respiratory tract infection. Risk factors examined were age, tobacco use, number and type of pre-existing comorbidities, emergency surgery, and number and type of surgeries. Bivariate and multivariable logistic regression models were conducted to identify risk factors for developing a PPC.

**Results:**

The PPC rate was 7.8%. Of the 418 hospitalizations in which a patient developed a PPC, the most common type of PPC was pneumonia (52.4%) and the mortality rate related to the PPC was 11.7%. Significant risk factors for a PPC were increasing age, greater number of comorbidities, emergency surgery, greater number of general surgeries, and abdominal surgery.

**Conclusions:**

PPCs are common in general surgery patients in low- and middle-income countries, with similar rates observed in high-income countries. These complications worsen patient outcomes and increase mortality. Quality improvement initiatives that employ resource-conscious methods are needed to reduce PPCs in low- and middle-income countries.

## Introduction

General surgery patients often have numerous comorbidities making them medically complex to manage and predisposing them to developing a postoperative pulmonary complication (PPC) [1]. PPCs contribute significantly to increased patient morbidity and 30-day mortality, prolonged hospital stays, and greater resource consumption, which can financially burden the hospital and patient [2–5]. Currently, PPCs are one of the most common postoperative complications in high-income countries (HICs), affecting approximately 10% of patients undergoing non-cardiac surgeries and up to 22% of patients considered high-risk due to surgery type or concomitant comorbid conditions [2, 6].

PPCs are conditions that impact the respiratory tract and compromise a patient’s clinical state following surgery [5, 7]. The severity of PPCs varies from minor complications to conditions that have serious implications on patient outcomes, such as pneumonia or acute respiratory distress syndrome [1, 8]. The causes of PPCs involve the interplay of patient- and surgery-related factors during the perioperative period [1, 2, 9]. General anesthesia lays the foundation for PPC development by affecting normal lung and chest wall physiology [2, 5, 9–12]. These pulmonary changes cause a restrictive lung pattern from atelectasis and promote secretion retention, predisposing patients to PPCs [2].

Certain patient- and surgery-related factors compound a general surgery patient’s risk for developing a PPC. Patient-specific features such as advanced age of 50 years and older, functional dependence, American Society of Anesthesiologists (ASA) class ≥ 2, congestive heart failure, and chronic obstructive pulmonary disease are well supported PPC risks [2, 9]. Surgical techniques, including laparoscopy, and surgical location near the diaphragm or respiratory tract confer significant PPC risk, regardless of a patient’s preoperative physical status [1, 2, 9]. Additionally, emergency surgery has been independently associated with PPC development because patients cannot be medically optimized beforehand [2, 9, 13, 14].

Although PPCs have been well studied and documented in HICs, little is known about PPCs and their risk factors in many low- and middle-income countries (LMICs). This gap is primarily attributed to the absence of electronic medical record databases that would enable PPC data analysis [15]. Validated PPC risk calculators and quality improvement initiatives have been developed in HICs and address risks significant in a HIC setting [16]. Disease processes, such as human immunodeficiency virus (HIV) and tuberculosis, that are prevalent in LMICs have not been carefully examined in current PPC research; yet, they complicate surgical outcomes and may represent important PPC risks in LMICs [17, 18].

South Africa is a LMIC with significant rates of HIV and tuberculosis [17–19]. Grey’s Hospital, located in the province of KwaZulu-Natal (KZN) in South Africa, is uniquely equipped with a Hybrid Electronic Medical Registry containing large volumes of clinical information useful for examining PPCs [20]. This public hospital residing in KZN’s capital of Pietermaritzburg is one of the province’s largest trauma centers and is an ideal location to investigate a lower-resource healthcare setting [21]. Identifying specific PPC risk factors at this hospital would guide PPC prevention to improve patient outcomes and reduce healthcare costs in LMICs.

The aim of this study was to examine the incidence of PPCs and their associated risk factors in adult general surgery patients in KZN, South Africa. A retrospective secondary data analysis was used to identify patient- and surgery-related factors associated with PPC development among general surgery patients at Grey’s Hospital, which could then be used to develop quality improvement (QI) initiatives tailored to prevent PPCs in high-risk patients in a LMIC healthcare setting.

## Materials and methods

### Design and setting

This study was a retrospective secondary analysis of data from adult patients admitted to the general surgery service at Grey’s Hospital from January 1, 2013 to December 31, 2017 (five years). The absence/presence of a PPC, number of PPCs, and types of PPCs during each general surgery hospitalization were determined. Risk factors examined were age, tobacco use, number and type of pre-existing comorbidities, and emergency surgery as well as number and type of surgeries.

Ethics approval for this study and maintenance of the registry were granted by the Biomedical Research Ethics Committee of the University of KwaZulu-Natal (reference number: BE 207/09 BCA 221/13). Institutional Review board (IRB) exemption was obtained by the University IRB in the United States (IRB protocol number: Pro00093495). The data extracted from the Hybrid Electronic Medical Registry (HEMR) database at Grey’s Hospital for this study were fully de-identified prior to the start of the secondary analysis. Due to the retrospective nature of this study, the Ethics Committee of the University of KwaZulu-Natal did not require informed consent of the participants included in the analysis.

Grey’s Hospital is a tertiary level public hospital in the KZN province, South Africa. The hospital has 530 beds and is part of the Pietermaritzburg Metropolitan Trauma Service, serving a population of over three million people [22]. Data were extracted from the hospital’s HEMR, a sophisticated relational database maintained by hospital residents and physicians. The HEMR data analyzed originated from three databases: General Surgery, Operations, and Morbidity. Patient records were merged by into a single dataset for the purpose of coding and deriving analysis variables. Data coding consisted of reviewing quantitative and qualitative data within each general surgery hospitalization record. For patients with multiple general surgery admissions during the five-year period, each hospital admission date was treated as a general surgery hospitalization. The final step was to create a final summary dataset with one record for each general surgery hospitalization.

**Fig 1:**
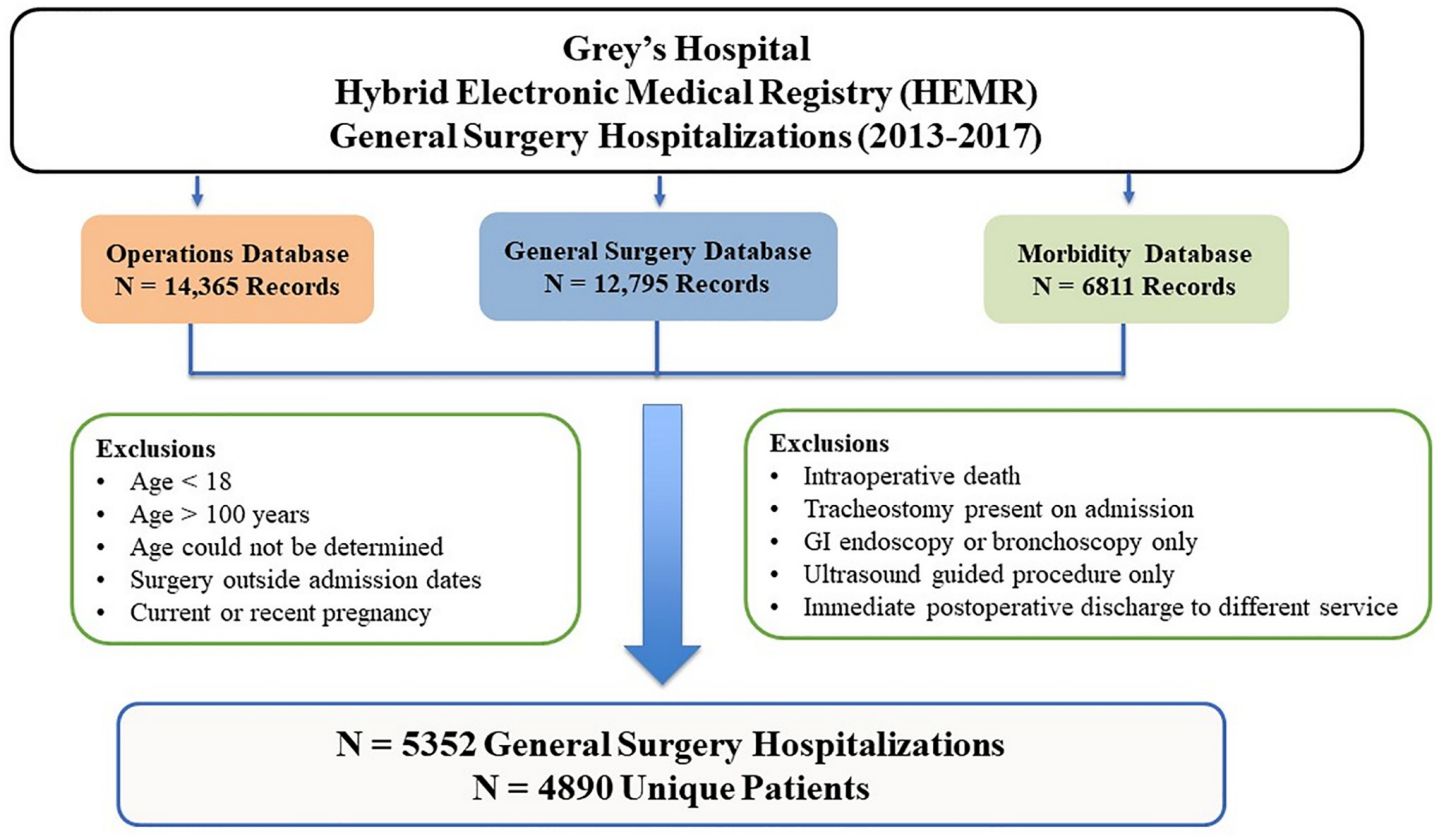
Determination of final analysis sample.

Only hospitalizations for adult patients (aged 18 to 100 years) with a general surgery admission during the five-year period were included. A hospitalization was excluded if the patient had any of the following: age could not be determined; tracheostomy on admission; surgery performed outside admission dates; intraoperative death; current or recent pregnancy; only a gastrointestinal endoscopy, bronchoscopy, or ultrasound-guided procedure performed; or patient was discharged to another service intraoperatively or immediately postoperatively. The final analysis sample was comprised of 5352 general surgery hospitalizations, representing 4890 unique patients (Fig 1).

### Measures

Analysis variables and their values were obtained from quantitative and qualitative data (clinical text fields) in the HEMR that was reviewed and coded to detail sample characteristics, risk factors, and outcomes. Dichotomous variables were coded as absent (0) or present (1). No documented evidence of a characteristic or outcome was coded as absent.

#### Patient characteristics

Patient characteristics were age, lifetime history of tobacco use, and 13 pre-existing comorbidities. Gender was not available in the database. The 13 comorbidities considered were 10 conditions linked to PPC development [1, 2, 5, 9] as well as two conditions (HIV and pulmonary tuberculosis) that have a high prevalence rate in South Africa [23]. The comorbid conditions were: acute kidney injury (AKI), anemia, asthma, cerebrovascular accident (CVA), chronic kidney disease (CKD), congestive heart failure (CHF), coronary artery disease (CAD), chronic obstructive pulmonary disease (COPD), diabetes mellitus (DM), hypertension (HTN), liver disease, pulmonary tuberculosis (TB), and retroviral disease (RVD, included HIV and/or retroviral disease). Total number of the 13 comorbidities present was also derived.

#### Surgery characteristics

The general surgeries during the hospital admission were categorized into 10 types: (1) abdominal; (2) thoracic; (3) vascular; (4) wound; (5) neurology; (6) urology; (7) orthopedic; (8) ear, nose, and throat (ENT); (9) gynecology (GYN); and (10) general surgery-other types, defined as any other type of surgery performed on the external portion of the trunk or perineum. The total number of general surgeries performed as well as the total number of types of surgery conducted during the hospitalization were also determined. The need for an emergency surgery during the general surgery hospitalization was also coded.

#### Postoperative pulmonary complications (PPCs)

The primary outcome was the development of a PPC during the general surgery hospitalization. A PPC was defined at least one of the following types of PPC: (1) pneumonia, (2) atelectasis, (3) acute respiratory distress syndrome (ARDS), (4) pulmonary edema, (5) prolonged ventilation, (6) pulmonary embolism (PE), (7) hemothorax, (8) pneumothorax, and (9) respiratory other, where respiratory other encompassed empyema, aspiration, pleural effusion, bronchopleural fistula, and lower respiratory tract infection [20]. The absence/presence of each type of PPC and total number of types of PPCs were also determined during the hospitalization. A patient who received a tracheostomy secondary to a PPC only was also determined; however, a tracheostomy was not included in the calculation of the total number of PPC types.

#### Mortality

Patient mortality was defined as a patient death for any reason during their hospitalization following a surgery. Mortality related to a PPC was defined as a patient death that was a direct result of a PPC during their hospitalization following a surgery.

### Data analysis

The analysis was conducted using SAS version 9.4 software (Cary, NC). Non-directional statistical tests with significance set at 0.05 for each test were conducted. Bivariate logistic regression was used to identify patient and surgery characteristics associated with an increased PPC risk among the 5352 general surgery hospitalizations. Risk factors significant at the 0.05 level were retained for the multivariable logistic regression model. Odds ratios (ORs) for the bivariate regression and adjusted ORs (aORs) for the multivariable regression along with their 95% confidence intervals (CIs) were used to address clinical significance of the risk factors. The sample size of 5352 hospitalizations provided at least 80% statistical power for the bivariate and multivariable logistic regression to examine risk factors for development of a PPC.

## Results

### Patient characteristics

Table 1 details the patient characteristics for each hospitalization. The mean patient age was 47.5 years (range: 18 to 100), with 26.8% of the patient being age 60 or older. Tobacco use was reported in 22.5%. The most common comorbidities were HTN (32.6%), followed by RVD (21.8%), and DM (16.0%). Pre-existing comorbidities ranged from 0 to 6, with 8.4% having three or more of comorbidities. The majority of the general surgery hospitalizations had a singular admission (91.8%), but number of admissions per patient ranged up to five (0.1%) over the five-year span.

**Table 1:** Patient characteristics for hospital admissions (N = 5352).

Characteristic	n (%)
**Age, in years**	47.5 (17.2%)
Age 18–39	1934 (36.1%)
Age 40–59	1987 (37.1%)
Age 60–79	1272 (23.8%)
Age 80–100	159 (3.0%)
**Tobacco use**	1205 (22.5%)
**Comorbidities**	
Hypertension (HTN)	1746 (32.6%)
Retroviral Disease (RVD)	1167 (21.8%)
Diabetes Mellitus (DM)	857 (16.0%)
Chronic Kidney Disease (CKD)	317 (5.9%)
Tuberculosis (TB)	297 (5.6%)
Asthma	147 (2.8%)
Anemia	134 (2.5%)
Coronary Artery Disease (CAD)	126 (2.4%)
Congestive Heart Failure (CHF)	103 (1.9%)
Cerebrovascular Accident (CVA)	102 (1.9%)
Acute Kidney Injury (AKI)	101 (1.9%)
Chronic Obstructive Pulmonary Disease (COPD)	46 (0.9%)
Liver Disease	25 (0.5%)
**Total Comorbidities**	
No comorbidities	2165 (40.5%)
One comorbidity	1779 (33.2%)
Two comorbidities	961 (18.0%)
Three or more comorbidities	447 (8.4%)

Total comorbidities = Number of 13 comorbidities present (observed range: 0 to 6)

### Surgical characteristics

Table 2 summarizes the surgical procedures per hospitalization. Almost 21% had at least one emergency surgery. The most common surgeries performed were abdominal (62.5%) and general surgery-other type (13.0%). In 98.1% of hospitalizations, only one type of surgery was performed. Four patients (1.9%) had two types of surgeries and one patient (0.02%) had three types of surgeries during the hospital admission. The total number of surgeries per hospitalization ranged from 1 to 8, with 1.8% having 3 or more surgeries. The number of abdominal surgeries ranged from 0 to 8, with 6.8% having two or more surgeries of this type.

**Table 2:** General surgeries during the hospitalization (N = 5352).

General Surgeries	n (%)
**One or more emergency surgeries**	1113 (20.8%)
**Types of surgeries**	
Abdominal	3343 (62.5%)
General surgery–other types	697 (13.0%)
Orthopedic	519 (9.7%)
Wound	393 (7.3%)
Ears Nose Throat (ENT)	300 (5.6%)
Vascular	135 (2.5%)
Thoracic	39 (0.7%)
Gynecology	22 (0.4%)
Urology	9 (0.2%)
Neurology	0 (0.0%)
**Number of surgery types**	
One type of surgeries performed	5248 (98.1%)
**Number of surgeries**	
One surgery	4830 (90.3%)
Two surgeries	425 (4.9%)
Three or more surgeries (range: 3 to 8)	97 (1.8%)
**Total abdominal surgeries**	
No abdominal surgery	2009 (37.5%)
One abdominal surgery	2981 (55.7%)
Two or more abdominal surgeries (range: 2 to 8)	362 (6.8%)

### PPCs

Table 3 describes the PPC rates for the 5352 hospitalizations and for the 418 patients who developed a PPC during their hospitalization. The PPC rate was 7.8% for the 5352 general surgery hospitalizations over the five-years, and the most common type of PPC was pneumonia (4.1%). Among the 418 hospitalizations in which the patient developed a PPC, most (89.0%) patients experienced one PPC type and 3.1% required a tracheostomy secondary to their PPC.

**Table 3:** Postoperative pulmonary complications during a hospitalization.

Postoperative Pulmonary Complication (PPC)	All Hospitalizations N = 5352 n (%)	Hospitalizations with a PPC N = 418 n (%)
**Any PPC event**	418 (7.8%)	418 (100%)
**PPC Type**		
Pneumonia	219 (4.1%)	219 (52.4%)
Prolonged ventilation	66 (1.2%)	66 (15.8%)
Respiratory other	60 (1.1%)	60 (14.4%)
Atelectasis	45 (0.8%)	45 (10.8%)
Acute Respiratory Distress Syndrome (ARDS)	30 (0.6%)	30 (7.2%)
Pulmonary embolism (PE)	24 (0.5%)	24 (5.7%)
Pulmonary edema	19 (0.4%)	19 (4.6%)
Pneumothorax	9 (0.2%)	9 (2.2%)
Hemothorax	0 (0.0%)	0 (0.0%)
**Number of types of PPCs**		
No type of PPC	4934 (92.2%)	---
1 type of PPC	372 (7.0%)	372 (89.0%)
2 types of PPCs	38 (0.7%)	38 (9.1%)
3 types of PPCs	8 (0.2%)	8 (1.9%)
**Tracheostomy Secondary to a PPC**	---	13 (3.1%)

#### Mortality

Almost 5% of the patients admitted for a general surgery died before discharge, while approximately 1% of the patients resulted in a death related to a PPC (Table 4). Among the 418 hospitalizations in which a patient developed a PPC, the mortality rate was 27.8% and the mortality rate resulting from a PPC was 11.7%. Among the 264 patients who died, 18.6% experienced death that was determined to be directly related to a PPC.

**Table 4:** In-hospital mortality outcomes.

Mortality During Hospitalization	All Hospitalizations N = 5352 n (%)	Hospitalizations with a PPC N = 418 n (%)	Hospitalizations with Mortality N = 264 n (%)
Mortality for any reason	264 (4.9%)	116 (27.8%)	---
Mortality related to a PPC	49 (0.9%)	49 (11.7%)	49 (18.6%)

### PPC risk factors: Bivariate regression

Tables 5 and 6 summarize the bivariate logistic regression results. Risk of developing a PPC was significantly associated with increasing age (p < .0001) and number of pre-existing comorbidities (p = 0.0002) as well as the presence of HTN (p = 0.0252), TB (p = 0.0093), CHF (p = 0.0013), and AKI (p < .0001). The PPC rate was 17.6% among those age 80 to 100 years compared to 5.4% among those age 18 to 39 years (OR = 3.8, p < .0001). Patients with 3 or more comorbidities (OR = 1.9, p < .0001) or 2 comorbidities (OR = 1.4, p = 0.0141) had a significantly higher risk of developing a PPC than those with no comorbidities; however, this was not the case for patients with one comorbidity (OR = 1.1, p = 0.5791).

**Table 5:** Risk factors for development of a PPC: Bivariate logistic regression results (N = 5352).

Factor	N	PPC Rate n (%)	OR	95% CI	p-value
**Age, in years**					**< .0001**
Age 80 to 100	159	28 (17.6%)	3.8	2.4–5.9	**< .0001**
Age 60 to 79	1272	134 (10.5%)	2.1	1.6–2.7	**< .0001**
Age 40 to 59	1987	152 (7.7%)	1.5	1.1–1.9	**0.0042**
Age 18 to 39 *(ref)*	1934	104 (5.4%)	---	---	---
**Tobacco use**					0.4728
Yes	1205	100 (8.3%)	1.1	0.9–1.4	0.4728
No *(ref)*	4147	318 (7.7%)	---	---	---
**# of comorbidities**					**0.0002**
Three or more	447	55 (12.3%	1.9	1.4–2.7	**< .0001**
Two	961	89 (9.3%)	1.4	1.1–1.9	**0.0141**
One	1779	128 (7.2%)	1.1	0.8–1.4	0.5791
None *(ref)*	2165	146 (6.7%)	---	---	---
**HTN**					**0.0252**
Yes	1746	157 (9.0%)	1.3	1.0–1.6	
No *(ref)*	3606	261 (7.2%)	---	---	
**RVD**					0.6981
Yes	1167	88 (7.5%)	1.0	0.7–1.2	
No *(ref)*	4185	330 (7.9%)	---	---	
**DM**					0.0699
Yes	857	80 (9.3%)	1.3	1.0–1.6	
No *(ref)*	2295	338 (7.5%)	---	---	
**CKD**					0.8717
Yes	317	24 (7.6%)	1.0	0.6–1.5	
No *(ref)*	5035	394 (7.8%)	---	---	
**TB**					**0.0093**
Yes	297	35 (11.8%)	1.6	1.1–2.4	
No *(ref)*	5055	383 (7.6%)	---	---	
**Asthma**					0.8689
Yes	147	12 (8.2%)	1.1	0.6–1.9	
No *(ref)*	5205	406 (7.8%)	---	---	
**CHF**					**0.0013**
Yes	103	17 (16.5%)	2.4	1.4–4.1	
No *(ref)*	5249	401 (7.6%)	---	---	
**AKI**					**< .0001**
Yes	101	24 (23.8%)	3.8	2.4–6.1	
No *(ref)*	5251	394 (7.5%)	---	---	

**Table 6:** Risk factors for development of a PPC: Bivariate logistic regression results (N = 5352).

Factor	N	PPC Rate n (%)	OR	95% CI	p-value
**Emergency surgery**					**< .0001**
Yes	1113	162 (14.6%)	2.7	2.2–3.3	
No *(ref)*	4239	256 (6.0%)	---	---	---
**Total surgeries**					**< .0001**
3+ surgeries	97	38 (39.2%)	9.7	6.3–14.8	**< .0001**
2 surgeries	425	78 (18.4%)	3.4	2.6–4.4	**< .0001**
1 surgery *(ref)*	4830	302 (6.3%)	---	---	---
**Abdominal surgery**					**< .0001**
Yes	3343	323 (9.7%)	2.2	1.7–2.7	
No *(ref)*	2009	95 (4.7%)	---	---	---
**# abdominal surgeries**					**< .0001**
2+ surgeries	362	103 (28.5%)	8.0	5.9–10.9	**< .0001**
1 surgery	2981	220 (7.4%)	1.6	1.3–2.1	**0.0002**
None *(ref)*	2009	95 (4.7%)	---	---	---
**General surgery-other** *					**< .0001**
Yes	697	16 (2.3%)	0.2	0.2–0.4	
No *(ref)*	4655	402 (8.6%)	---	---	---
**Orthopedic surgery**					0.1959
Yes	519	33 (6.4%)	0.8	0.5–1.1	
No *(ref)*	4833	385 (8.0%)	---	---	---
**Wound surgery**					0.3602
Yes	393	26 (6.6%)	0.8	0.5–1.2	
No *(ref)*	4959	392 (7.9%)	---	---	---
**ENT surgery** *					**0.0133**
Yes	300	12 (4.0%)	0.5	0.3–0.9	
No *(ref)*	5052	406 (8.0%)	---	---	---
**Vascular surgery** *					**0.0146**
Yes	135	1 (0.7%)	0.1	0.0–0.6	
No *(ref)*	5217	417 (8.0%)	---	---	---

N = Number with the characteristics specified in the row; PPC rate = number (n) and percent (%) with a PPC among those with the characteristics specified in the row (N); OR = Odds ratio, CI = Confidence Interval; total surgeries = total number of surgeries performed during the hospitalization;

*Less than 5% of hospitalizations with the surgery type have a PPC

Increased PPC risk was significantly associated with an emergency surgery (p < .0001), greater number of surgeries (p < .0001), an abdominal surgery (p < .0001), and greater number of abdominal surgeries (p < .0001). The PPC rate was 14.6% for emergency surgeries compared to 6.0% for those without an emergency (OR = 2.7). The PPC rate was 6.3% with one surgery, 18.4% with two surgeries, and 39.2% with three surgeries. Of note, the risk of a PPC was almost 10 times higher in hospitalizations in which the patient had 3 or more surgeries compared to those with one surgery (OR = 9.7). The PPC rate was 9.7% among those with an abdominal surgery compared to 4.7% for those without abdominal surgery (OR = 2.2); and the risk was significantly higher among those with 2 or more abdominal surgeries compared to those with no abdominal surgery (OR = 8.0, p < .0001).

Interestingly, the risk of a PPC was significantly lower in patients who received the following types of surgery when compared those who did not receive the surgery: other types of general surgery (p < .0001), ENT (p = 0.0133), and vascular surgery (p = 0.0146). Of note, the number of patients receiving either an ENT or vascular surgery was small, therefore these results should be interpreted with caution.

### PPC risk factors: Multivariable regression

Risk factors significant at the 0.05 level in the bivariate regression were considered for the multivariable logistic regression. The multivariable model (Table 7) included six risk factors: (1) age, (2) total number of comorbidities, (3) emergency surgery, (4) number of surgeries, (5) abdominal surgery, and (6) TB. The analysis focused on number of comorbidities instead of specific comorbidities, apart from TB due to its higher prevalence in South Africa. The dichotomized abdominal surgery factor rather than number of abdominal surgeries was included since the latter was highly correlated with total number of surgeries. Finally, we omitted other types of general surgery, ENT surgery, and vascular surgery because less than 5% of the hospitalizations with the surgery type had a PPC and each of these surgery types were associated with a lower risk of PPCs in the bivariate analysis.

**Table 7:** Risk factors for PPCs: Multivariable logistic regression results (N = 5352).

Factor	aOR	aOR 95% CI	p-value
**Age, in years**			**< .0001**
Age 80 to 100	6.4	3.9–5.9	**< .0001**
Age 60 to 79	3.1	2.3–4.1	**< .0001**
Age 40 to 59	1.9	1.1–2.5	**< .0001**
Age 18 to 39 *(ref)*	---	---	---
**# of comorbidities**			**0.0059**
Three or more comorbidities	1.9	1.3–2.7	**0.0008**
Two comorbidities	1.3	1.0–1.8	0.0875
One comorbidity	1.1	0.8–1.4	0.5986
No comorbidities *(ref)*	---	---	---
**Emergency surgery**			**< .0001**
Yes	2.0	1.6–2.6	
No *(ref)*	---	---	---
**Total number of surgeries**			**< .0001**
3+ surgeries	7.5	4.8–11.9	**< .0001**
2 surgeries	2.5	1.9–3.4	**< .0001**
1 surgery *(ref)*	---	---	---
**Abdominal surgery**			**< .0001**
Yes	2.1	1.6–2.7	
No *(ref)*	---	---	---
**Pulmonary tuberculous (TB)**			0.0694
Yes	1.5	1.0–2.3	
No *(ref)*			

aOR = adjusted odds ratios; 95% CI = 95% confidence interval for the aOR

Five of the six factors in the model were significantly related to the development of a PPC, after adjusting for all other factors. Risk of a PPC was associated with increasing age (p < .0001). Compared to the 18 to 39 age group, the odds of a PPC were almost two times greater in the 40 to 59 age group (aOR = 1.9, p < .0001), three times greater in the 60 to 79 age group (aOR = 3.1 p < .0001), and six times greater in the 80 to 100 age group (aOR = 6.4, p = < .0001). The PPC risk was nearly two times greater among those with three or more comorbidities compared to those with no comorbidity (aOR = 1.9, p = 0.0008) and two times higher for those patients needing an emergency surgery compared to those who did not (aOR = 2.0, p < .0001). Those with multiple surgeries were at much greater risk for a PPC. PPC risk was seven times higher among patients with three or more surgeries (aOR = 7.5, p < .0001) and two times greater among those with two surgeries (aOR = 2.5, p < .0001) compared to those with one surgery. PPC risk was significantly higher among those with an abdominal surgery (aOR = 2.1, p < .0001). Finally, PPC risk tended to be higher in those with a history of TB (aOR = 1.5, p = 0.0694).

## Discussion

The goal of this secondary analysis was to gain a better understanding of PPCs and their risk factors among general surgery patients in a LMIC. Currently, the PPC literature stems from HICs with risk factors that may not be generalizable to LMICs. LMICs generally lack resources that are standard in a HIC and this can create disparities spanning from preoperative patient optimization to postoperative enhanced recovery.

Risk stratification for pulmonary complications in general surgical patients can be a useful tool for providers in making perioperative decisions. Identifying at-risk patients can reduce morbidity and mortality as well as reduce the burden of healthcare associated costs and resources [1]. This is particularly valuable to LMICs where resources can be limited.

The PPC incidence rate of 7.8% among general surgical patients observed in this study was consistent with current studies reporting a PPC incidence rate from 2%-19% in noncardiac surgery patients [4, 5, 6, 24]. Our findings support that the KZN region has risk factors for PPCs that are in accordance with literature from HICs including advanced age, comorbidities, and surgical sites in the upper abdomen or thorax. Our findings also demonstrated some variation in PPC risks unique to and prevalent in LMICs, such as pulmonary TB [9, 25].

PPCs contribute importantly to perioperative morbidity and mortality [16]. Literature from HICs report PPC associated mortality rates ranging from 8–24% [14]. Our results indicated that among patients who died in the hospital, almost 19% died because of a PPC. Additionally, nearly 12% of the patients who developed a PPC died during their hospitalization as a direct result of their PPC. These outcomes are consistent with other studies, which have found PPC development to be associated with mortality, particularly mortality within 30-days [9, 24, 25]. While our analysis was limited to in-hospital mortality, these results indicate that PPCs play an important role in perioperative outcomes and PPC prevention is necessary to reduce patient mortality and improve health outcomes.

The most common type of PPC reported was pneumonia, affecting over half of the hospitalizations in which a PPC developed. This concurs with studies identifying pneumonia as a common post-surgical PPC [5, 6]. Atelectasis, the most common PPC type reported in the literature [6], was only indicated in 10.8% of hospitalizations that developed a PPC. This could be due to the lack of postoperative chest x-rays for confirmation or underreporting in the HEMR. Since atelectasis has been associated with the secondary development of pneumonia, atelectasis may not have been fully captured prior to the development of pneumonia [26].

Increasing age was a risk factor for PPC development, which is consistent with the prior research. Advanced age has been shown to be an important independent predictor for PPCs, with older age categories conferring higher PPC risk [9, 24]. Physiologic aging leads to reductions in respiratory muscle strength, parenchymal elasticity, and alveolar surface area [7]. These changes increase respiratory work, ventilation/perfusion mismatch, and decrease the efficacy of coughing which can contribute to atelectasis, aspiration and pneumonia [7].

Tobacco use, particularly, more than 1 pack per day, has been linked to PPCs in previous studies [9, 25], but was not a risk factor in our results. The lack of a relationship may be partially attributed to incomplete reporting regarding tobacco history. Number of comorbidities was a risk factor for PPC development. Overall health status is an important factor for pulmonary risk and is commonly stratified using ASA classification [16]. Studies have demonstrated that ASA class 2 patients have an increased risk of PPCs, with higher ASA class associated with increased PPC risk [7, 9]. Although the HEMR did not include ASA class, number of comorbidities is a possible surrogate to evaluate overall patient health status. Interestingly, patients with two or more of the selected comorbid conditions would qualify for an ASA class ≥ 2 [16].

Types of comorbidities related to PPCs offered important similarities and differences with the existing literature. For example, CHF is an established PPC risk factor [9, 25], and this relationship was also observed in this study. Although HTN has not been directly linked to PPCs in the literature, HTN was a risk factor associated with PPCs in our findings. Further, HTN was the most prevalent comorbidity in our sample, which aligns with other LMIC studies [17].

Pulmonary TB and HIV were examined due to their high prevalence rates in South Africa and potential pathophysiologic consequences during the perioperative period. Although TB treatment is highly effective, many patients experience ongoing pulmonary impairment from the infection such as pulmonary fibrosis or COPD [27] and require greater perioperative attention [18]. Although TB history was associated with PPC emergence in the bivariate analysis, this relationship was diminished after controlling for other risk factors. Interestingly, RVD, which includes HIV and other retroviral diseases, was not a significant risk factor for PPCs.

Several surgical factors were associated with developing a PPC. Number of general surgeries was a major risk factor in our findings. The PPC rate was highest among patients undergoing abdominal surgery, which is consistent with prior research reporting upper abdominal surgery as a PPC predictor [24]. Emergency surgeries were also associated with increased PPC risk. Preoperative optimization before emergency surgery is challenging and these patients warrant aggressive pulmonary protection in the postoperative period.

### Limitations

Free text fields included in the HEMR provided detailed clinical information, but extensive review and data coding were required to develop the variables and quantify data needed to address the aims. Furthermore, the lack of systematic documentation in free text fields may have resulted in underreporting and underrepresentation of clinically important information for this retrospective secondary analysis. This concern is particularly pertinent to atelectasis, the most common PPC type reported in the previous studies [6]. The atelectasis rate in our study was lower than reported in the published studies conducted in HICs. Thus, it is possible that atelectasis as well as non-acute pneumonia were not adequately documented in the HEMR.

### Future quality improvement initiatives

The most common PPC was pneumonia. Several evidence-based practice interventions have successfully reduced pneumonia rates in HICs. Interventions include early mobilization, limiting the use of nasogastric tubes when possible, hand hygiene, utilizing ventilator associated pneumonia checklists, and revised sedation practices [28–30]. Protocols including head of bed elevation >30 degrees, oral hygiene, aspiration prophylaxis, and ventilator weaning trials have historically decreased pneumonia rates from 12% to 0% [28].

The implementation of a verified PPC risk calculator to identify high-risk patients may be useful in general surgery patients. There are several pre-existing PPC risk calculators available, namely the ARISCAT calculator and Arozullah Respiratory Failure Risk Index [24, 31], that could help identify high-risk patients in a real-time setting. A perioperative PPC prevention program coupled with the implementation of a risk calculator could maximize PPC reduction. Moore and colleagues [29] reported a decrease in PPCs and length of stay with the implementation of an Enhanced Recovery After Surgery Plus (ERAS+) protocol coupled with the ARISCAT calculator in a study on postoperative lung expansion with incentive spirometry, patient mobilization, and oral hygiene, which all require minimal to no additional resources.

## Conclusions

PPCs still continue to be one of the most common adverse events after surgery and can significantly increase patient morbidity, mortality, and use of hospital resources. Although South Africa may have some similarities in risk factors from published research in HICs, there are important conditions that could affect patients in LMIC that should be critically considered for optimal patient care. Preventing PPCs is crucial and may be achieved through identifying the patients at an increased risk for developing a PPC and implementing a lung protection protocol for postoperative care. Further investigation is required to determine the feasibility and effectiveness of these strategies among the general surgery patient population.

## Supporting information

S1 Dataset (CSV)

S1 File (DOCX)
