# Supplementary material for: Identification of risk factors for postoperative pulmonary complications in general surgery patients in a low-middle income country
Source: PLoS One. 2022 Oct 11;17(10):e0274749. doi: 10.1371/journal.pone.0274749 (PMC9553039; doi:10.1371/journal.pone.0274749)
Supplement: S1 File — (DOCX) [file pone.0274749.s002.docx]

**PLOS Dataset Codebook**

| **Variable Name** | **Label** | **Coding** | **Definition/description** |
| --- | --- | --- | --- |
| **subid** | Subject ID | Numeric | Subject ID – anonymous ID number |
| **encounter_new** | encounter | Unique General Surgery Admission | General Surgery encounter (hospitalizations) - distinct unrelated hospitalization number after exclusions applied. |
| age2 | Age, in years | Continuous  18-89 | Age in years of patient at admission |
| age_cat8 | Age: 8 categories | Categorical | Age in years of patient at admission divided into 8 categories and includes: 18-29, 30-39, 40-49, 50-59, 60-69, 70-79, 80-89, and 90-100 |
| age_cat4 | Age: 4 categories | Categorical | Age in years of patient at admission divided into 4 categories and includes: 18 to 39, 40-59, 60-79, and 80-100 |
| tobacco_f | Tobacco use | No=0  Yes=1 | Presence of pre-existing tobacco use, determined on admission |
| total_comorbidities_f | Total number of comorbidities | Continuous | Total number of pre-existing 13 comorbidities present during each hospital admission; Sum of (1) asthma_f, (2) copd_f, (3) cad_f, (4) htn_f, (5) cva_f, (6) chf_f, (7) tb_f, (8) rvd_f, (9) diabetes_f, (10) anemia_f, (11) liver_f, (12) acute kidney_f, (13) chronic kidney_f. Each individual condition is coded as 0=No, 1=Yes |
| total_comorbidities_cat_f | Total comorbidities: 4 categories | Categorical | Total number of the 13 comorbidities present during each hospital admission (above) divided into 4 categories: 0= 0 comorbidities, 1= 1 comorbidity, 2= comorbidities, and 3= 3 or more comorbidities |
| asthma_f | Asthma | No=0  Yes=1 | Presence of pre-existing asthma as a comorbidity |
| copd_f | Chronic Obstructive Pulmonary Disease | No=0  Yes=1 | Presence of pre-existing COPD disease as a comorbidity |
| cad_f | Coronary Artery Disease | No=0  Yes=1 | Presence of pre-existing coronary artery disease as a comorbidity |
| htn_f | Hypertension | No=0  Yes=1 | Presence of pre-existing hypertension as a comorbidity |
| cva_f | Cerebrovascular Accident | No=0  Yes=1 | Presence of pre-existing cerebrovascular accident history as a comorbidity |
| chf_f | Congestive Heart Failure | No=0  Yes=1 | Presence of pre-existing congestive heart failure as a comorbidity |
| tb_f | Tuberculosis | No=0  Yes=1 | Presence of pre-existing tuberculosis, not treated, as a comorbidity |
| hiv_f | Human Immunodeficiency Virus | No=0  Yes=1 | Presence of pre-existing Human Immunodeficiency Virus (HIV) as a comorbidity |
| orvd_f | Other Retroviral Disease | No=0  Yes=1 | Presence of pre-existing retroviral disease (nonspecific) as a comorbidity |
| rvd_f | HIV or Retroviral Disease | No=0  Yes=1 | Presence of pre-existing retroviral disease or HIV as a comorbidity; Yes=1 defined as hiv_f=1 or orvd_f=1 |
| diabetes_f | Diabetes | No=0  Yes=1 | Presence of pre-existing diabetes mellitus as a comorbidity |
| anemia_f | Anemia | No=0  Yes=1 | Presence of pre-existing anemia as a comorbidity |
| liver_f | Liver Disease | No=0  Yes=1 | Presence of pre-existing liver disease as a comorbidity |
| acute_kidney_f | Acute Kidney Injury | No=0  Yes=1 | Presence of pre-existing acute kidney injury as a comorbidity |
| chronic_kidney_f | Chronic Kidney Disease | No=0  Yes=1 | Presence of pre-existing chronic kidney disease as a comorbidity |
| obesity_f | Obesity | No=0  Yes=1 | Presence of pre-existing obesity as a comorbidity |
| gensurg |  | No=0  Yes=1 | All are gensurg = 1;  Identifies the analysis samples: Hospitalizations who met the eligibility criteria for the GenSurg PPC analysis in 2021 |
| gen_abd_surg_01_f | General Abdominal Surgery | No=0  Yes=1 | General surgery was performed in the abdomen or to the abdominal organs. Includes: surgery to the GI tract (stomach, small intestine, and large intestine), surgery to the liver, or surgery to the kidney |
| gen_abd_surg_f | General Abdominal Surgery | Continuous | Total number of surgeries performed in the abdomen or to the abdominal organs. Includes surgery to the GI tract (stomach, small intestine, and large intestine), surgery to the liver, or surgery to the kidney |
| tenkoff_01_f | Tenkoff Catheter Placement | No=0  Yes=1 | If Tenkoff catheter placement was performed |
| tenkoff_f | Tenkoff Catheter Placement | Continuous | Total number of Tenkoff catheter placements performed |
| gen_abd_surg2_01_f | General Abdominal Surgery | No=0  Yes=1 | If general surgery was performed in the abdomen or to the abdominal organs, and includes: surgery to the GI tract (stomach, small intestine, and large intestine), surgery to the liver, surgery to the kidney, **and Tenkoff catheter placement.** |
| gen_abd_surg2_f | General Abdominal Surgery | Continuous | Total number of surgeries performed in the abdomen or to the abdominal organs, and includes: surgery to the GI tract (stomach, small intestine, and large intestine), surgery to the liver, surgery to the kidney, and **Tenkoff catheter placement** |
| gen_abd_surg2_cat02_f | General Abdominal Surgery:  2 categories | Categorical | Number of surgeries performed in the abdomen or to the abdominal organs, and includes: surgery to the GI tract (stomach, small intestine, and large intestine), surgery to the liver, surgery to the kidney, **and Tenkoff catheter placemen**t divided into 3 categories including 0=no abdominal surgeries, 1= abdominal surgery, and 2=2 or more abdominal surgeries |
| thoracic_surg_01_f | Thoracic Surgery | No=0  Yes=1 | If surgery was performed in the thorax or to the thoracic organs, and includes: surgery to the heart, surgery to the lungs, surgery to the diaphragm (if entered through thorax), surgery to blood vessels located in the thorax, or surgery involving opening of the thorax |
| thoracic_surg_f | Thoracic Surgery | Continuous | total number of surgeries performed in the thorax or to the thoracic organs, and includes: surgery to the heart, surgery to the lungs, surgery to the diaphragm (if entered through thorax), surgery to blood vessels located in the thorax, or surgery involving opening of the thorax |
| ENT_surg_01_f | ENT Surgery | No=0  Yes=1 | Surgery was performed to the ears, nose, throat, or neck |
| ENT_surg_f | ENT Surgery | Continuous | Total number of surgeries performed to the ears, nose, throat, or neck |
| wound_surg_01_f | Wound Surgery | No=0  Yes=1 | Surgery was performed in a wound. Includes: wound debridement, wound closure, wound wash out, skin grafting, or incision and drainage of an abscess. |
| wound_surg_f | Wound Surgery | Continuous | Total number of surgeries performed in a wound. Includes: wound debridement, wound closure, wound wash out, skin grafting, or incision and drainage of an abscess. |
| ortho_surg_01_f | Orthopedic Surgery | No=0  Yes=1 | Orthopedic surgery was performed to bones |
| ortho_surg_f | Orthopedic Surgery | Continuous | Total number of orthopedic surgeries performed to bones |
| Amputation_surg_01_f | Amputation Surgery | No=0  Yes=1 | Amputation surgery was performed |
| Amputation_surg_f | Amputation Surgery | Continuous | Total number of amputation surgeries performed |
| Ortho_surg2_01_f | Orthopedic Surgery | No=0  Yes=1 | Orthopedic surgery was performed to bones including amputation |
| Ortho_surg2_f | Orthopedic Surgery | Continuous | Total number of orthopedic surgeries performed to bones including amputation |
| uro_surg_01_f | Urology Surgery | No=0  Yes=1 | Surgery was performed to the urologic organs, and includes: procedures involving the bladder, ureters, or urethra |
| uro_surg_f | Urology Surgery | Continuous | Total number of surgeries performed to the urologic organs, and includes: procedures involving the bladder, ureters, or urethra |
| vasc_surg_01_f | Vascular Surgery | No=0  Yes=1 | If vascular surgery was performed to blood vessels |
| vasc_surg_f | Vascular Surgery | Continuous | Total number of vascular surgeries performed to blood vessels |
| neuro_surg_01_f | Neurology Surgery | No=0  Yes=1 | If neurologic surgery was performed and includes: surgery to the brain or surgery involving the skull |
| neuro_surg_f | Neurology Surgery | Continuous | Total number of neurologic surgeries performed and includes: surgery to the brain or surgery involving the skull |
| gyn_surg_01_f | Gynecology Surgery | No=0  Yes=1 | If surgery was performed to the female reproductive organs |
| gyn_surg_f | Gynecology Surgery | Continuous | Total number of surgeries performed to the female reproductive organs |
| Gen_surg_other_01_f | Other  General  Surgery | No=0  Yes=1 | If surgery was performed to the trunk that doesn’t include opening the thoracic or abdominal cavity |
| Gen_surg_other_f | Other  General  Surgery | Continuous | Total number of surgeries performed to the trunk that doesn’t include opening the thoracic or abdominal cavity |
| GI_endoscopy_01_f | GI Endoscopy | No=0  Yes=1 | If gastrointestinal endoscopic procedure was performed (not included as a surgery) |
| GI_endoscopy_f | GI Endoscopy | Continuous | Total number of gastrointestinal endoscopic procedures performed (not included in total number of surgeries or total number of surgery types) |
| ercp_01_f | Endoscopic Retrograde Cholangio-Pancreatography | No=0  Yes=1 | If Endoscopic Retrograde Cholangio-Pancreatography (ERCP) was performed (not included as a surgery) |
| ercp_f | Endoscopic Retrograde Cholangio-Pancreatography | Continuous | Total number of ERCPs performed (not included in total number of surgeries or total number of surgery types) |
| bronch_01_f | Bronchoscopy | No=0  Yes=1 | If bronchoscopy was performed (not included as a surgery) |
| bronch_f | Bronchoscopy | Continuous | Total number of bronchoscopies performed (not included in total number of surgeries or total number of surgery types) |
| ultrasound_guided_surg_01_f | Ultrasound Guided Procedure | No=0  Yes=1 | If ultrasound guided procedure was performed (not included as a surgery) |
| ultrasound_guided_surg_f | Ultrasound Guided Procedure | Continuous | Total number of ultrasound guided procedures performed (not included in total number of surgeries or total number of surgery types) |
| total_types_surgeries_f | Total Number of Types of Surgeries | Continuous | Total number of surgery types performed per encounter that includes general abdominal surgery, thoracic surgery, ENT surgery, wound surgery, orthopedic surgery, urologic surgery, vascular surgery, neurologic surgery, gynecologic surgery, and other general surgery |
| total_num_surgeries_f | Total Number of Surgeries | Continuous | Total number of surgeries performed per encounter that includes general abdominal surgery, thoracic surgery, ENT surgery, wound surgery, orthopedic surgery, urologic surgery, vascular surgery, neurologic surgery, gynecologic surgery, and other general surgery;  Derived from summing:  gen_abd_surg2_f, thoracic_surg_f, ent_surg_f , wound_surg_f, ortho_surg2_f, uro_surg_f, vasc_surg_f, neuro_surg_f, gyn_surg_f and gen_surg_other_f |
| total_num_surgeries_cat13_f | Total Number of Surgeries: Categories 1,2,3+ | Categorical | Total number of surgeries performed per encounter that includes general abdominal surgery, thoracic surgery, ENT surgery, wound surgery, orthopedic surgery, urologic surgery, vascular surgery, neurologic surgery, gynecologic surgery, and other general surgery divided into 3 categories including 1=1 surgery, 2=2 surgeries, and 3=3 or more surgeries |
| total_num_surgeries_cat14_f | Total Number of Surgeries: Categories 1,2,3,4+ | Categorical | Total number of surgeries performed per encounter that includes general abdominal surgery, thoracic surgery, ENT surgery, wound surgery, orthopedic surgery, urologic surgery, vascular surgery, neurologic surgery, gynecologic surgery, and other general surgery divided into 4 categories including 1=1 surgery, 2=2 surgeries, 3=3 surgeries and 4=4 or more surgeries |
| emergency_tag_f | Emergency Surgery | No=0  Yes=1 | Emergent surgery was performed during the admission |
| total_types_ppc | Total Number of Respiratory Events | Continuous | Total number of postoperative pulmonary complications for each patient. Oncludes: pneumonia/atelectasis, acute respiratory distress syndrome (ARDS)/pulmonary edema, pulmonary embolism (PE)/respiratory other morbidity, prolonged ventilation, hemothorax, or pneumothorax; Yes=1, defined as  pna_f=1 or atel_f=1 or ards_f=1 or pulm_edem_f=1 or pe_f=1 or resp_oth_f=1 or prolonged_vent_f=1 or hemothorax_f=1 or pneumothorax_f=1 |
| ppc_tag_f | Postoperative Pulmonary Complications | No=0  Yes=1 | Presence of any post-operative pulmonary complication, and includes: pneumonia/atelectasis, acute respiratory distress syndrome (ARDS)/pulmonary edema, pulmonary embolism (PE)/respiratory other morbidity, prolonged ventilation, hemothorax, or pneumothorax; Yes=1, defined as pneumonia_atel_sum=1 or  pna_f=1 or atel_f=1 or  ards_pulm_f=1 or ards_f=1 or pulm_edem_f=1 or pe_f=1 or resp_oth_f=1 or prolonged_vent_f=1 or  pneumothorax_hemothorax_f=1 or hemothorax_f=1 or pneumothorax_f=1; No=0, defined as pneumonia_atel_sum=0 or  pna_f=0 or atel_f=0 or  ards_pulm_edem_f=0 or ards_f=0 or pulm_edem_f=0 or pe_f=0 or resp_oth_f=0 or prolonged_vent_f=0 or  pneumothorax_hemothorax_f=0 or hemothorax_f=0 or pneumothorax_f=0 |
| mortality_f | Mortality | No=0  Yes=1 | Death before discharge occurred (in-hospital mortality) |
| mortality_ppc_f | PPC Mortality | No=0  Yes=1 | Death before discharge occurred as a result of a PPC |
| atel_pna_f | Pneumonia and atelectasis | No=0  Yes=1 | Combines the development of pneumonia or atelectasis as a morbidity; Yes=1, defined as pneumonia_atel_f=1 or pna_f=1 or atel_f=1; No=0, defined as pneumonia_atel_f=0 and pna_f=0 and atel_f=0  Note: Variables were combined due to their similarities in pathophysiology |
| pna_f | Pneumonia | No=0  Yes=1 | Development of pneumonia as a morbidity |
| atel_f | Atelectasis | No=0  Yes=1 | Development of atelectasis as a morbidity |
| ards_pulm_edem_f | Acute respiratory distress syndrome (ARDS) and pulmonary edema | No=0  Yes=1 | Combines the development of acute respiratory distress syndrome (ARDS) or pulmonary edema as a morbidity; Yes=1, defined as ards_pulm_edem_f=1 if ards_f=1 or pulm_edem_f=1; No=0, defined as ards_pulm_sum=0 and ards_sum=0 and pulm_edem_sum=0  (Variables were combined due to their similarities in pathophysiology) |
| ards_f | ARDS | No=0  Yes=1 | Development of Acute Respiratory Distress Syndrome as a morbidity after a surgery |
| pulm_edem_f | Pulmonary edema | No=0  Yes=1 | Development of pulmonary edema as a morbidity after a surgery |
| pe_f | Pulmonary embolism | No=0  Yes=1 | Development of a pulmonary embolism as a morbidity after a surgery |
| resp_oth_f | Other Respiratory Morbidity | No=0  Yes=1 | Presence of other respiratory morbidity, and includes: empyema, aspiration, pleural effusion, bronchopleural fistula, or lower respiratory tract infection |
| prolonged_vent_f | Prolonged Ventilation | No=0  Yes=1 | Development of the need for prolonged ventilation as a morbidity after a general surgery. Also includes: patients with respiratory failure, pulmonary collapse, or remaining intubated postoperatively |
| hemothorax_f | Hemothorax | No=0  Yes=1 | Development of a hemothorax as a morbidity after a surgery |
| pneumothorax_f | Pneumothorax | No=0  Yes=1 | Development of a pneumothorax as a morbidity after a surgery |
| pneuomothorax_hemothorax_f | Pneumothorax and Hemothorax | No=0  Yes=1 | Combines the development of pneumothorax or hemothorax as a morbidity; Yes=1, defined as;  pneumothorax_f=1 or hemothorax_f=1 THEN pneuomothorax_hemothorax_f=1; No=0, defined as pneumothorax_f=0 and hemothorax_f=0 then pneuomothorax_hemothorax_f=0  Note: Variables were combined due to their similarities in pathophysiology) |
| trach_morbidity_f | Tracheostomy from Morbidity | No=0  Yes=1 | The presence of a tracheostomy as the result of a PPC after a respiratory morbidity (not a PPC) |
| trach_admission_f | Tracheostomy on Admission | No=0  Yes=1 | The presence of a tracheostomy NOT due to a respiratory morbidity |
